# Leave-one-out-analysis (LOOA): web-based tool to predict influential proteins and interactions in aggregate-crosslinking proteomic data

**DOI:** 10.6026/973206300200004

**Published:** 2024-01-31

**Authors:** Nirjal Mainali, Meenakshisundaram Balasubramaniam, Jay Johnson, Srinivas Ayyadevara, Robert J. Shmookler Reis

**Affiliations:** 1 Bioinformatics Program, University of Arkansas for Medical Sciences and University of Arkansas at Little Rock, Little Rock, AR, 72205, USA; 2 Department of Geriatrics, Reynolds Institute on Aging, University of Arkansas for Medical Sciences, Little Rock, AR, 72205, USA; 3 McClellan Veterans Medical Center, Central Arkansas Veterans Healthcare Service, Little Rock, AR, 72205, USA

## Abstract

Many age-progressive diseases are accompanied by (and likely caused by) the presence of protein aggregation in affected tissues. Protein aggregates are conjoined by complex protein-protein interactions, which remain poorly understood. Knowledge of the proteins that comprise aggregates, and their adherent interfaces, can be useful to identify therapeutic targets to treat or prevent pathology, and to discover small molecules for disease interventions. We present web-based software to evaluate and rank influential proteins and protein-protein interactions based on graph modelling of the cross linked aggregate interactome. We have used two network-graph-based techniques: Leave-One-Vertex-Out (LOVO) and Leave-One-Edge-Out (LOEO), each followed by dimension reduction and calculation of influential vertices and edges using Principal Components Analysis (PCA) implemented as an R program. This method enables researchers to quickly and accurately determine influential proteins and protein-protein interactions present in their aggregate interactome data.

## Background:

Protein aggregation is a hallmark of most age-related diseases including Alzheimer's disease (AD), cardiovascular disease (CVD), Parkinson's disease (PD), Huntington's disease (HD), and many other age-progressive diseases. In the case of AD, protein aggregation is thought to be initiated by "seed" proteins, amyloid beta (Aβ) and hyper phosphorylated tau (hP-tau) [1]. As these oligomers expand, they recruit other proteins that transiently unfold or misfold, chiefly through hydrophobic interactions with amyloid or neurofibrillary tangles (NFTs) [2, 3]. Several tools have been made available that help in analyzing proteomics data [4,5, 6,7]. We have previously shown that analysis of crosslinking data, obtained by permeating aggregates with small "click reagents", allows us to identify and quantify protein-protein interactions in an aggregate (i.e., the aggregate interactome). We first followed this strategy for cultured neuroblastoma cells bearing a familial-AD double mutation (SY5Y-APPSw) [8]. We analyzed interactome data from crosslinked aggregates to rank influential proteins based on the total number of interactions in the SY5Y-APPSw interactome. Relative to wild-type neuroblastoma cells (SY5Y-WT), SY5Y-APPSw aggregate proteins have far higher connectivity. We now show that each protein's influence can be predicted based only on the topology of the aggregate interactome, through a novel approach based on graph modeling.

In order to more fully utilize the cross linked aggregate-interactome data, we developed a web-based tool to prioritize proteins (vertices or nodes) and protein-protein interactions (edges) in the SY5Y-APPSw interactome, by their predicted influence on the complexity (degree sum) of the aggregate network. We have primarily used R programming to develop two programs: Leave-One-Vertex-Out (LOVO) and Leave-One-Edge-Out (LOEO) analyses. LOVO analysis deletes one vertex at a time and calculates the influence of that node, as the factor by which its deletion reduces the total complexity, Σ (all node degrees) of the interactome. LOEO instead deletes one edge at a time and calculates edge influence in the same way. The influence is then considered as a function of diverse network descriptors such as Degree, Eigenvector Value, Betweenness, Closeness, and Clustering Coefficient. Calculating and accounting for the influence of vertices and edges provides insights into the roles of proteins and their interactions in aggregate formation and stability, and also helps to identify candidate targets for drugs that act as protein-protein interaction inhibitors (PPII) to reduce aggregate burden, and thus to ameliorate diseases that feature protein aggregation. Therefore, it is of interest to describe a Leave-One-Out-Analysis (LOOA) web-based tool to predict influential proteins and interactions in aggregate-crosslinking proteomic data.

## Methodology:

## Usage:

**Figure 1:**
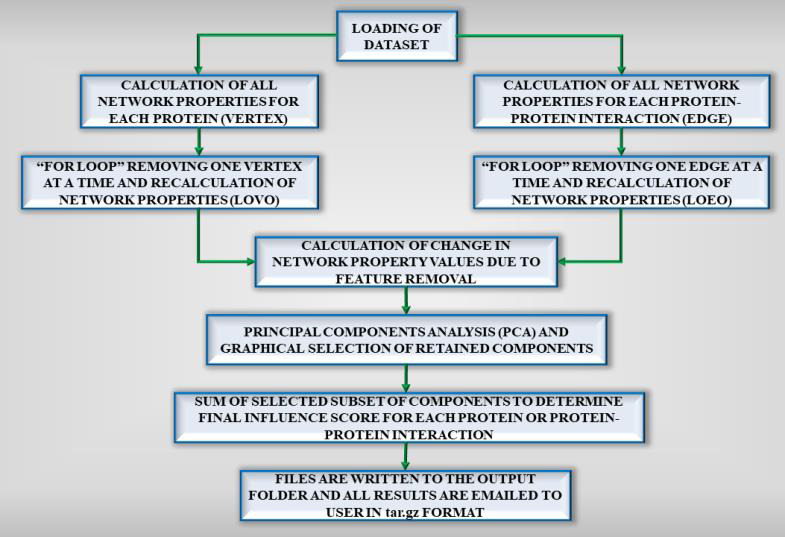
Workflow for LOOA design and development

**Figure 2:**
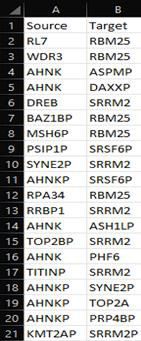
Input data structure. Data are entered in .csv format, comprising Source and Target columns, with source proteins and target proteins as values from SY5Y-APPSw aggregate-interactome data.

The web server for conducting Leave-One-Out-Analysis is provided under Online Tools in https://simlab.uams.edu. The basic workflow of this tool is explained in Figure 1. It takes input in Comma Separated Value (.csv) format. The input file must have Source and Target as its column headers, with the list of aggregate proteins as row values (Figure 2). There are five interaction properties that are calculated for LOVO and LOEO, which jointly characterize the interactome connectivity increment contributed by each vertex or edge.

## Degree Centrality (DC):

The Degree Centrality of a vertex is the number of edges or interactions it has in the network graph. The higher the degree of a vertex or node implies greater DC indicating its influence.

## Eigenvector Centrality (EC):

Eigenvector Centrality is an algorithm for network graphs that measures the transitive influence of the vertex. Vertices with high Eigenvector Centrality score are connected to many other vertices which themselves have high EC scores.

## Betweenness Centrality (BC):

Betweenness Centrality measures the extent to which a vertex lies in the path between other vertices. Higher BC scores connote higher influence of a node in the network, by conjoining other vertex clusters; thus, removal of a high-BC vertex will disrupt assembly of large aggregates.

## Closeness Centrality (CC):

Closeness Centrality measures the average remoteness of a vertex from all other vertices, calculated as the sum of the inverse of distances. Vertices with high CC score have relatively short distances to all other vertices, enabling efficient spread of information through the network, and contribute to their influence.

## Global Clustering Coefficient (GCC)

GCC differs from other centrality properties; it is a measure of the density of triangles in a network. It measures the extent to which vertices in a graph tend to cluster together. Global clustering coefficients are used in both LOVO and LOEO and are based on triplets (or triangle) of nodes. Closed triplets occur when there are three vertices (A, B, C) connected to each other, forming three edges (A-B, B-C, and C-A) and a closed triangle, whereas "open triplets" are those in which three vertices are connected, but form only two edges (e.g., A-B and B-C). The global clustering coefficient is calculated as the number of closed triplets divided by the total number of triplets (open or closed) in the graph.

Aggregate proteins are uploaded as shown in Figure 2, and LOVO and LOEO are conducted. Five network properties (NP) are calculated, and recalculated upon sequential removal of individual vertices or edges; influence scores are calculated as ΔNP = NP_initial_ - NP_minus-protein-i._ PCA reduces the dimensionality of NP inputs, yielding a composite influence score for each analysis. Aggregate interactome data for SY5Y-APPSw cells [8] illustrate the procedures involved.

**Figure 3:**
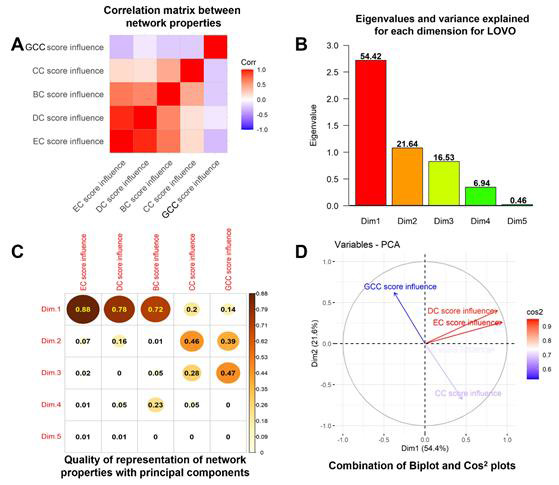
Principal components analysis (PCA) after Leave One Vertex Out (LOVO) analysis. (A) Correlation matrix summarizing the correlations among individual network descriptors, ranging from very high positive correlation (red) to very high inverse correlation (blue). White signifies that two variables show little or no correlation. (B) Bar plot showing top 5 dimensions/principal components with their eigenvalues; numbers over bars indicate %-variance explained. (C) Cos^2^ correlation plot showing the quality of representation of each network property (NP) with each principal component (PC or dimension, Dim). (D) Biplot and Cos^2^ plots are combined to form a "hybrid plot" showing correlations between network properties and quality of representation of each property by two components (indicated by arrow length and their Cos^2^ values represented by color (see inset key).

[1] Figure 3A shows a LOVO matrix of correlations between NP changes after removal of each vertex. Influences by EC, DC and BC are inter-correlated, while GCC is unrelated to other network properties.

[2] Principal Components Analysis (PCA) reduces dimensionality to yield composite scores. N Components are selected based on Kaiser's rule (eigenvalue ≥1) [9] OR variance explained ≥10%; exceeding either threshold allows inclusion. A histogram displays the eigenvalue for each dimension and its "%variance explained" (Figure 3B). Here, LOVO accepts three principal components, explaining 92% of total variance.

[3] Cos^2^ (squared cosine) summarizes relative representation of NPs by dimensions (calculated as PCA values squared). For LOVO, the Cos^2^ value of PC1 (Dim.1) is highly correlated to EC, DC, and BC scores, with Cos^2^ correlation values ≥0.72; while PC2 (Dim.2) has highest correlations to CC and GCC; and PC3 (Dim.3) has the highest correlation to GCC (Figure 3C). The combination of PC1, PC2, and PC3 thus represents all five properties. Omission of PC3 would have little effect, since GCC is well represented by PC2.

[4] A diagram combining biplot and Cos^2^ (Figure 3D) shows two positively correlated variables grouped together in the upper-right quadrant while negatively correlated variables lie on opposite quadrants. The representation of DC and EC in the first two components is greater than other scores, indicated by greater distance from the origin and larger Cos^2^ values.

**Figure 4:**
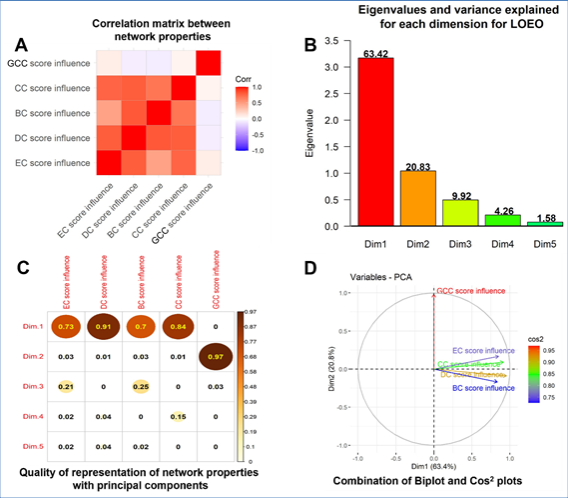
Principal components analysis (PCA) after Leave One Edge Out (LOEO) analysis. (A) Correlation matrix summarizing the correlations among individual network descriptors, as in Fig 2A. (B) Bar plot showing top 5 dimensions/principal components with their eigenvalues; numbers over bars indicate %variance explained. (C) Cos^2^ correlation plot showing quality of representation of each NP with each principal component (PC or Dim). (D) Biplot and Cos^2^ plots are combined to form a "hybrid plot" showing correlations between network properties and quality of representation of each property by two components (indicated by arrow length and their Cos^2^ values indicated by color (see inset).

[5] The LOEO correlation matrix (Figure 4A) reveals highly inter-correlated influences of EC, DC, and CC scores, while the GCC influence is unrelated to other NPs.

[6] PCA here indicates that the first two components have eigenvalues ≥1 and variances explained >10%, both dictating their selection; they together explain >84% of total variance (Figure 4B).

[7] Cos^2^ values of dimensions/components indicate that Dim.1/PC1 is correlated to EC, DC, BC, and CC scores >0.7, whereas Dim.2/PC2 has a near-perfect 0.97 correlation to GCC. Therefore, selection of the first two components is justified since the first two components together represent all NPs calculated (Figure 4C).

[8] The combined biplot/Cos^2^ plot (Figure 4D) shows EC, DC, CC, and BC clustered together, reflecting their high inter-correlation; they are well represented by Dim. 1, while GCC is largely orthogonal to other properties, and very well represented by Dim.2/PC2.

**Figure 5:**
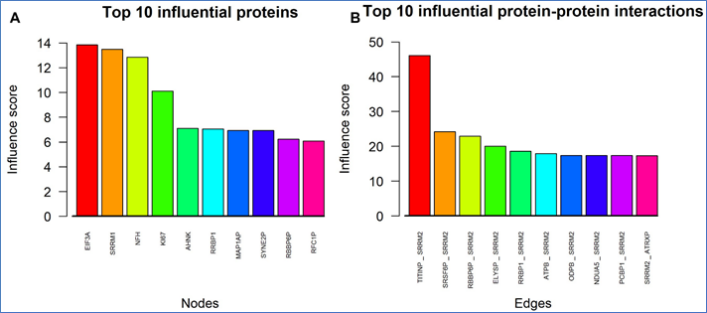
Top influential proteins and protein-protein interactions in SY5Y-APPSw aggregate interactome data. (A) Bar plot of top 10 influential vertices (nodes or proteins) after conducting LOVO analysis, sorted by combined influence scores. (B) Bar plot of top 10 influential protein-protein interactions after conducting LOEO analysis, sorted by combined influence scores.

[9] Histograms (Figure 5) show the top 10 influential proteins and interactions, after LOVO and LOEO respectively.

## Discussion:

The influence scores of all aggregate proteins and their protein-protein interactions are calculated based on LOVO and LOEO, with further characterization by PCA. We previously characterized influential proteins in SY5Y-APPSw aggregate-interactome data based only on degree and number of interactions of each protein [8]. We partitioned this aggregate interactome into 17 Mega-hubs (≥100 interactions), 77 Major hubs (50 - 99 interactions), 248 Midi-hubs (10 - 49 interactions) and 192 Mini-hubs (6 - 9 interactions). After conducting LOVO analysis, 13 out of 17 mega-hub proteins fell in the top 25 influential proteins, including numerous RNA-binding proteins such as EIF3A, SRRM1, DDX46P, SRSF6, TR140, and RBM25 (see ref. [10]). Other mega-hubs were centered on cell-cycle proteins such as AHNK (inhibitor of cell proliferation) and KI67 (which prevents aggregation of mitotic chromosomes), as well as PRC2C (stress granule assembly), SYNE2 (binds F-actin, tethers nucleus to cytoskeleton), RRBP1 (potassium homeostasis regulator), and RBBP6 (inhibitor of apoptosis). Of 77 major-hub proteins, 12 were among the top 25 influential proteins: MAP1A, RFC1, ZN638, NIPBL, RNPS1, SAFB1, TOP1, BAZ1A, KMT2A, HNRPR, BCLF1 and TRIPC, all of which were previously implicated in AD [11,12,13, 14]. Also, RNAi knockdowns of genes encoding EIF3A, SRSF6, RBBP6, ASPM, RFC1, and RNPS1 improved chemotaxis significantly in *C. elegans* strain CL2355, an AD model expressing human Aβ1-42 in all neurons leading to age-progressive or thermal-induction-dependent loss of normal chemo-attraction to n-butanol. Inclusion of over 50% of mega-hub proteins, and about 16% of major hub proteins, among the top 25 influential vertices predicted by LOVO, is consistent with a sharp drop in aggregate complexity upon removal of any one of these proteins. These observations support the premise that Leave-One-Vertex Out identifies influential proteins in the aggregate interactome. Influential protein-protein interactions predicted by Leave-One-Edge Out (LOEO) analysis highlights key interactions between influential vertices with mega-hub proteins, along with several major-hub proteins previously implicated in AD such as PRP8 (regulation of spliceosomes), ATRX (chromatin remodeling), ELYS (nuclear pore assembly), SPB1 (rRNA methylation), and PAIRB (proteasomal degradation and apoptosis) among the top 25 influential protein-protein interactions. This implies that disruption of these PPIs would reduce aggregate burden and may lead to the discovery of beneficial small molecules that disrupt these PPI interfaces in aggregates.

## Conclusions:

Leave-one-out-analyses, comprising Leave-One-Vertex-Out (LOVO) and Leave-One-Edge-Out (LOEO) modules, are efficient and useful computational methods to predict influential proteins and protein-protein interactions (respectively) once a complex aggregate interactome has been defined by cross-linking proteomics. This method is valuable for determining influential proteins and their interactions in any aggregate isolated from tissues, cells, or models of Alzheimer's, Parkinson's, Huntington's, or cardiovascular diseases. Formation and accrual of such aggregates are important diagnostic markers, and putative *causal agents*, for age-progressive diseases in general. The availability of these web-based tools, presented in a convenient and intuitive user interface, will help users to easily conduct these analyses, at any level of programming and data-analysis expertise. For example, the identification of influential proteins and interactions that participate in SY5Y-APPSw amyloid-aggregate interactomes [8] implicated proteins and PPIs that were either previously implicated in Alzheimer's disease or involved in important pathways impacting protein homeostasis. We expect that pursuit of these proteins is likely to contribute to better understanding of etiologic mechanisms that lead to Alzheimer's disease. These proteins may thus hold value as AD biomarkers and/or as therapeutic-intervention targets for prevention or amelioration of this devastating disease. We anticipate that broader use of these analytic/predictive computational tools will offer similar targets for intervention in other age-dependent diseases. Furthermore, the tools proposed here are capable of handling empirical protein-protein interaction data derived from other protein crosslinking methods, including formaldehyde and other crosslinking agents, and PPIs inferred from yeast two hybrid analyses, to implicate the most influential proteins observed in complex interactomes.
